# Bone Regeneration Following Implant Failure Using the Khoury Technique: A Case Report

**DOI:** 10.7759/cureus.111806

**Published:** 2026-06-30

**Authors:** Stefano Trasarti, Enrica Giammarinaro, Ugo Covani, Simone Marconcini

**Affiliations:** 1 Oral Surgery, Private Practice, Bolzano, ITA; 2 Oral Surgery, Istituto Stomatologico Toscano, Versilia General Hospital, Lido di Camaiore, ITA; 3 Oral Surgery, Saint Camillus International University of Health Sciences (UniCamillus), Rome, ITA

**Keywords:** alveolar ridge reconstruction, autogenous bone graft, esthetic zone, implant failure, khoury technique

## Abstract

Implant retreatment following implant failure represents a biologically and technically demanding scenario, particularly in the esthetic zone. Sites of previous implant failure are frequently characterized by residual inflammation, altered bone physiology, and reduced regenerative potential, which may negatively affect the prognosis of replacement implants. A staged, biologically driven approach is therefore recommended to restore hard and soft tissues prior to implant replacement. This clinical case report describes the management of a compromised anterior maxillary site following implant failure using the Khoury technique with exclusive autogenous bone. A young female patient presented with significant esthetic impairment caused by improper implant placement performed earlier, resulting in excessive gingival display and inadequate prosthetic conditions. After atraumatic implant removal and site debridement, three-dimensional bone reconstruction was performed using thin cortical bone plates harvested from the mandibular retromolar area to stabilize autogenous particulate bone, without the use of biomaterials or barrier membranes. Implant placement was performed four months after augmentation in regenerated vital bone, allowing correct three-dimensional prosthetic positioning. Soft tissue optimization was achieved using a connective tissue graft. The patient was followed clinically and radiographically for up to seven years after implant placement. The long-term outcomes demonstrated stable peri-implant hard and soft tissues, satisfactory esthetic integration, and maintenance of regenerated bone volume over time. Within the limitations of a single case report, this clinical presentation supports the use of a staged autogenous bone-based regenerative approach for the management of extensive bone defects following implant failure in sites with compromised local biology.

## Introduction

Implant failure is frequently associated with extensive peri-implant bone loss and compromised local biology, making retreatment challenging and more challenging than primary implant placement [1]. Sites of previous implant failure often present residual inflammation, reduced vascularization, altered bone physiology, and impaired wound healing, all of which negatively affect the prognosis of replacement implants [2]. In the esthetic zone, these challenges are further amplified by the need to restore both hard and soft tissue architecture while achieving prosthetically driven implant positioning. For single-tooth implant rehabilitation in the esthetic region, treatment success should not be assessed solely on the basis of implant survival or functional stability. Rather, it should encompass a combination of outcomes, including optimal functional performance, harmonious esthetic integration of the peri-implant soft tissues and prosthetic reconstruction, and positive patient-reported outcomes reflecting satisfaction with the treatment result.

Survival rates of replacement implants have been reported to be lower than those of initially placed implants, emphasizing the need for biologically driven regenerative strategies [3-5]. In cases of severe bone loss, particularly in the esthetic zone, a staged treatment approach is recommended to restore hard and soft tissues before implant replacement [6].

Various regenerative approaches have been proposed for the management of complex ridge defects, including guided bone regeneration with xenografts, allografts, and autogenous bone grafts [7]. Among these, autogenous bone grafting remains widely used because of its osteogenic, osteoinductive, and osteoconductive properties [8]. The Khoury technique utilizes thin cortical bone plates to stabilize particulate autogenous bone, promoting predictable three-dimensional regeneration with limited resorption [9-10]. To date, evidence supporting the use of the Khoury technique for the reconstruction of sites affected by implant failure is scarce. Most published studies have focused on horizontal ridge augmentation in non-failed sites, while only limited data are available on its effectiveness in retreatment following implant loss. This report describes the use of the Khoury technique to reconstruct a severely compromised anterior maxillary site following implant failure, with long-term clinical and radiographic follow-up.

## Case presentation

**Figure 1:**
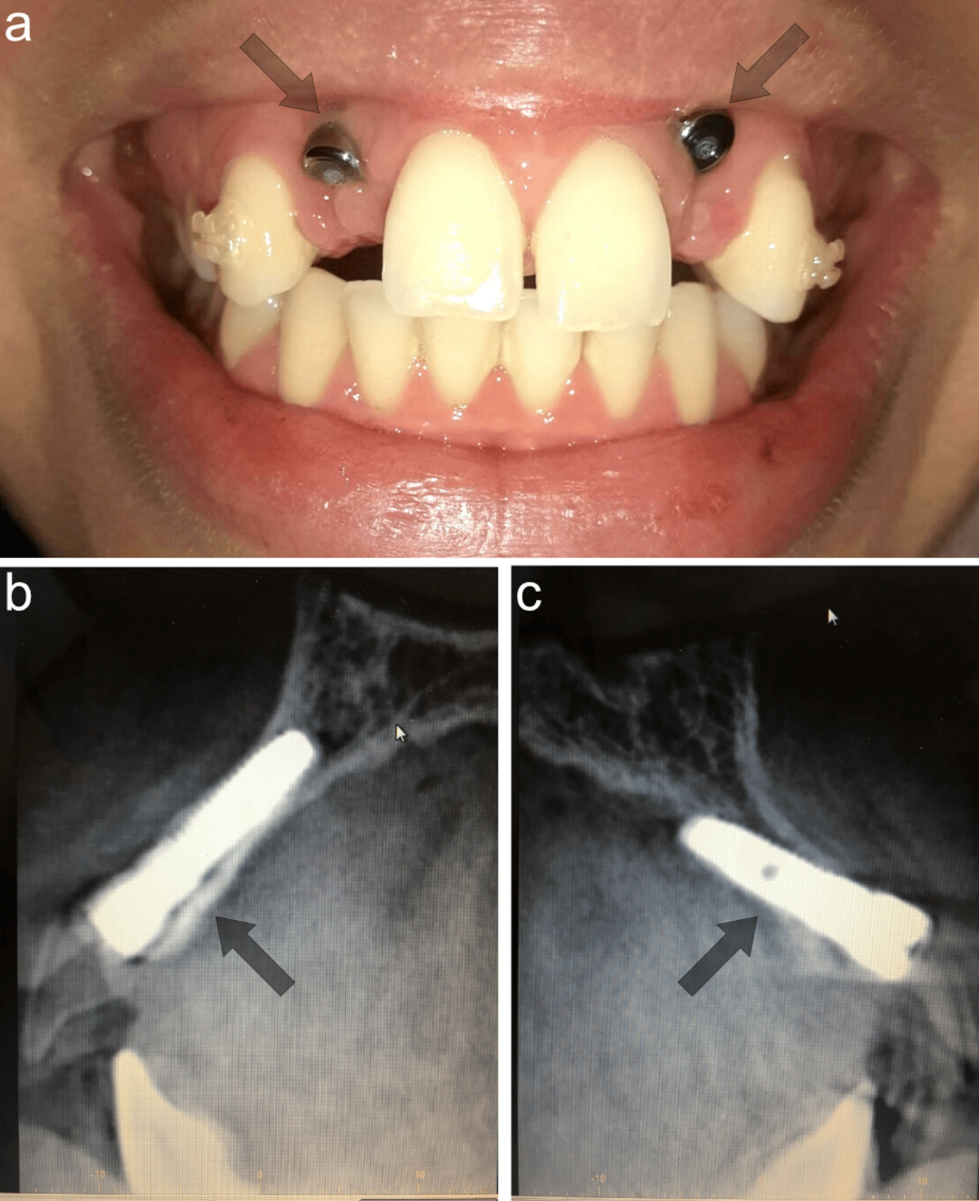
Baseline intraoral clinical view (a) and corresponding radiographic image showing implants located at sites 1.2 (b) and 2.2 (c) at the initial presentation.

**Figure 2:**
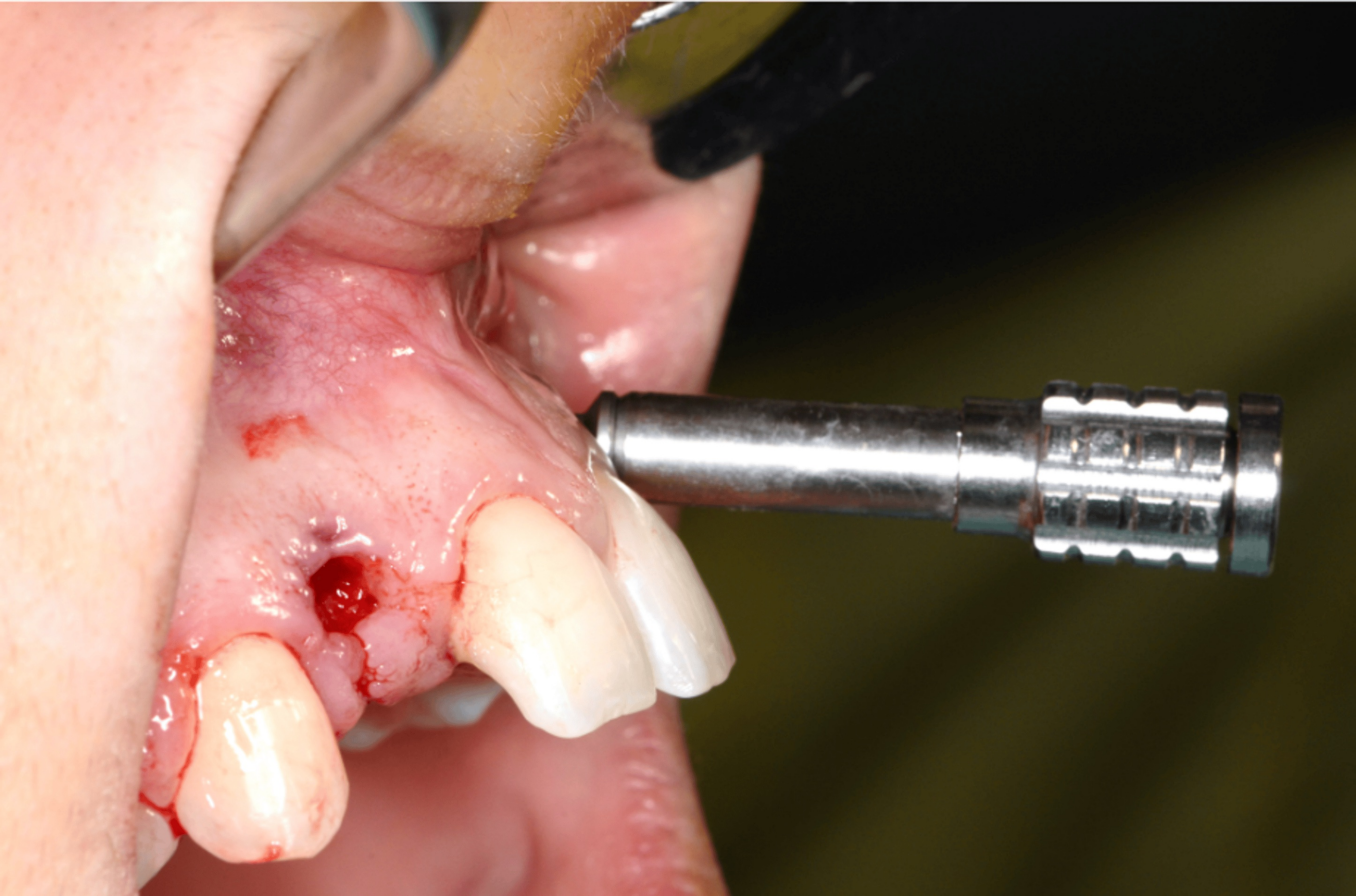
Evaluation of implant angulation revealing axial malposition of both implants prior to their removal.

A 20-year-old female patient presented in 2017 to a private practice in Bolzano, Italy, complaining of esthetic dissatisfaction in the maxillary anterior region. The patient exhibited a pronounced gingival display caused by improper implant placement in the lateral incisor sites (teeth 1.2 and 2.2), performed approximately three years earlier. Clinical and radiographic evaluation revealed severe implant malposition, inadequate peri-implant bone volume, and compromised esthetics (Figures 1, 2).

Based on the International Team for Implantology (ITI) SAC (Straightforward, Advanced, Complex) classification, the case was classified as advanced in terms of esthetic risk. After evaluating alternative treatment options, implant removal followed by staged reconstruction was selected.

Written informed consent for publication was obtained. Ethical approval was not required, as this was a retrospective case report conducted in accordance with the Declaration of Helsinki.

Clinical management

Implant Removal and Site Preparation

Systemic antibiotic therapy (amoxicillin/clavulanic acid, 2 g) was administered two hours preoperatively. Under local anesthesia, both implants were atraumatically removed, and thorough debridement of the sites was performed. Systemic antibiotic therapy was prescribed due to the presence of a previous peri-implant infection and extensive surgical manipulation

A cone-beam computed tomography (CBCT) scan obtained three months later demonstrated extensive vertical and horizontal bone deficiency on both vestibular and palatal aspects, indicating the need for three-dimensional bone reconstruction.

Bone Augmentation

**Figure 3:**
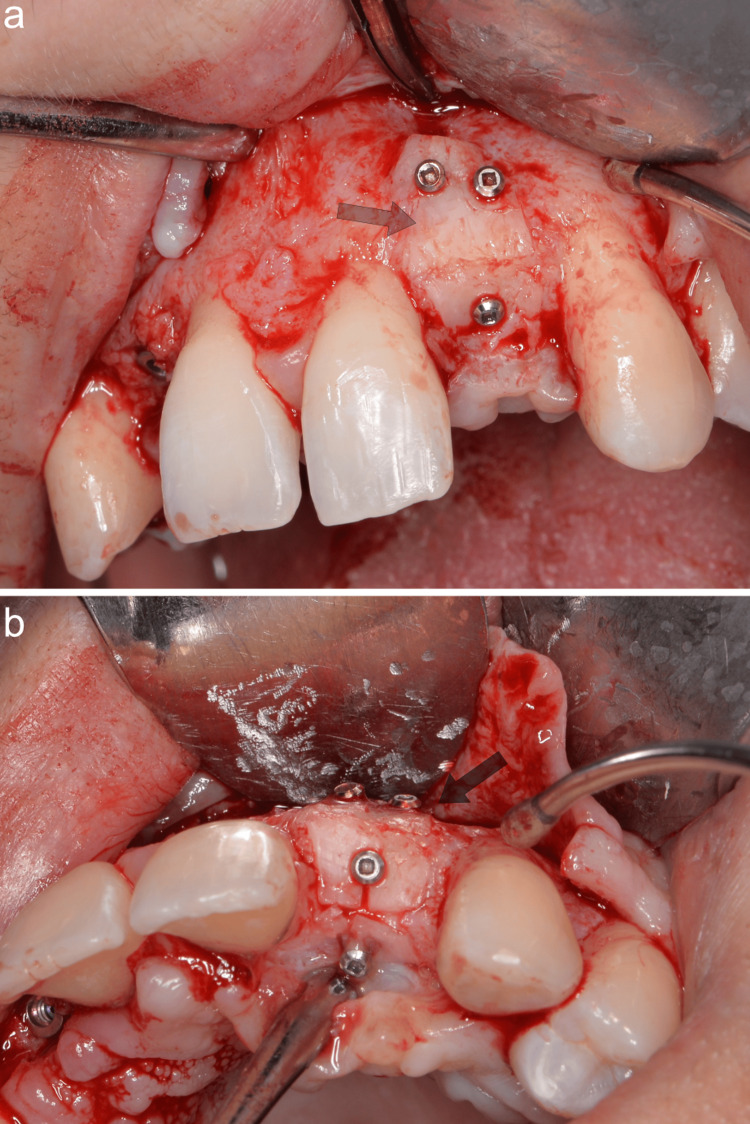
a) Intraoperative clinical view showing the fixation of two cortical bone laminae used for the reconstruction of the buccal and crestal aspects of the alveolar bone defect following implant removal (buccal aspect). b) Occlusal aspect.

A crestal incision was performed, revealing vertical defects ranging from 4 to 8 mm. Bone augmentation was carried out according to the Khoury technique. A mandibular retromolar bone block was harvested, split into three thin cortical plates, and fixed with microscrews to reconstruct the buccal and palatal walls of both implant sites (Figures 3a, 3b).

Two laminas were used at the 2.2 level and one in position 1.2. The interposed space was filled with autogenous particulate bone harvested using a bone scraper. No barrier membranes were used.

Approximately 8 mm of combined vertical and horizontal bone gain was achieved. No graft exposure occurred during healing.

Implant Placement and Prosthetic Phase

**Figure 4:**
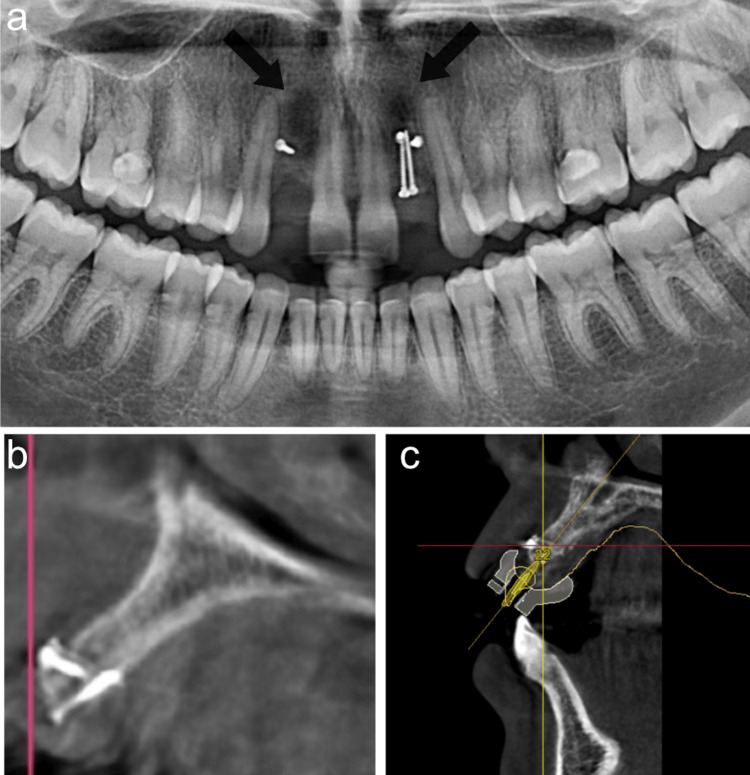
Postoperative radiographic image documenting the reconstructed alveolar bone after surgical healing: a) panoramic radiograph; b) and c) cross-sectional views.

Postoperative panoramic radiographs and CBCT imaging confirmed stable three-dimensional regeneration (Figure 4).

**Figure 5:**
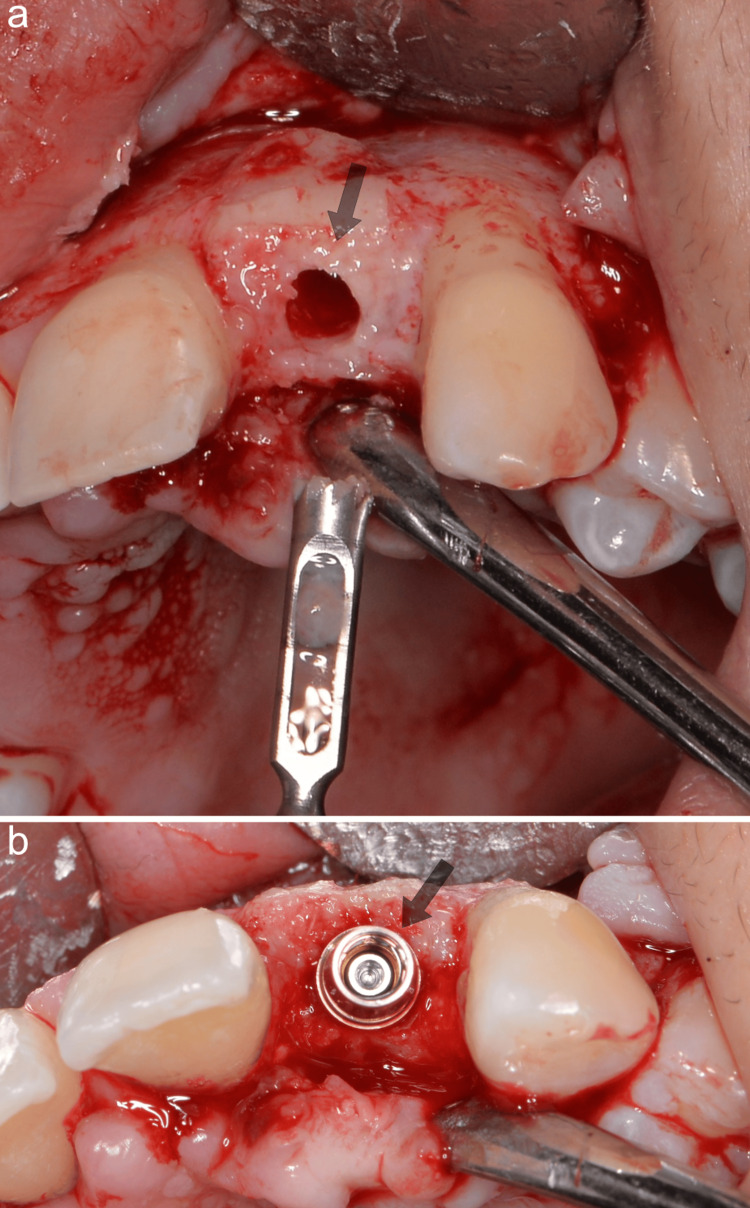
Intraoral clinical view of the reconstructed 2.2 site after healing (a) and the placement of new implant (b).

Thus, four months after augmentation, implants were placed in the correct three-dimensional prosthetic position, fully surrounded by regenerated vital bone (Figure 5).

Implants were uncovered three months later. A connective tissue graft was required at one site to optimize peri-implant soft tissue volume and quality. Provisional restorations were delivered after one month. Following six months of functional loading, orthodontic treatment was limited to closure of the interincisal diastema and did not affect the peri-implant tissues; definitive zirconia crowns were subsequently placed.

Clinical outcomes

**Figure 6:**
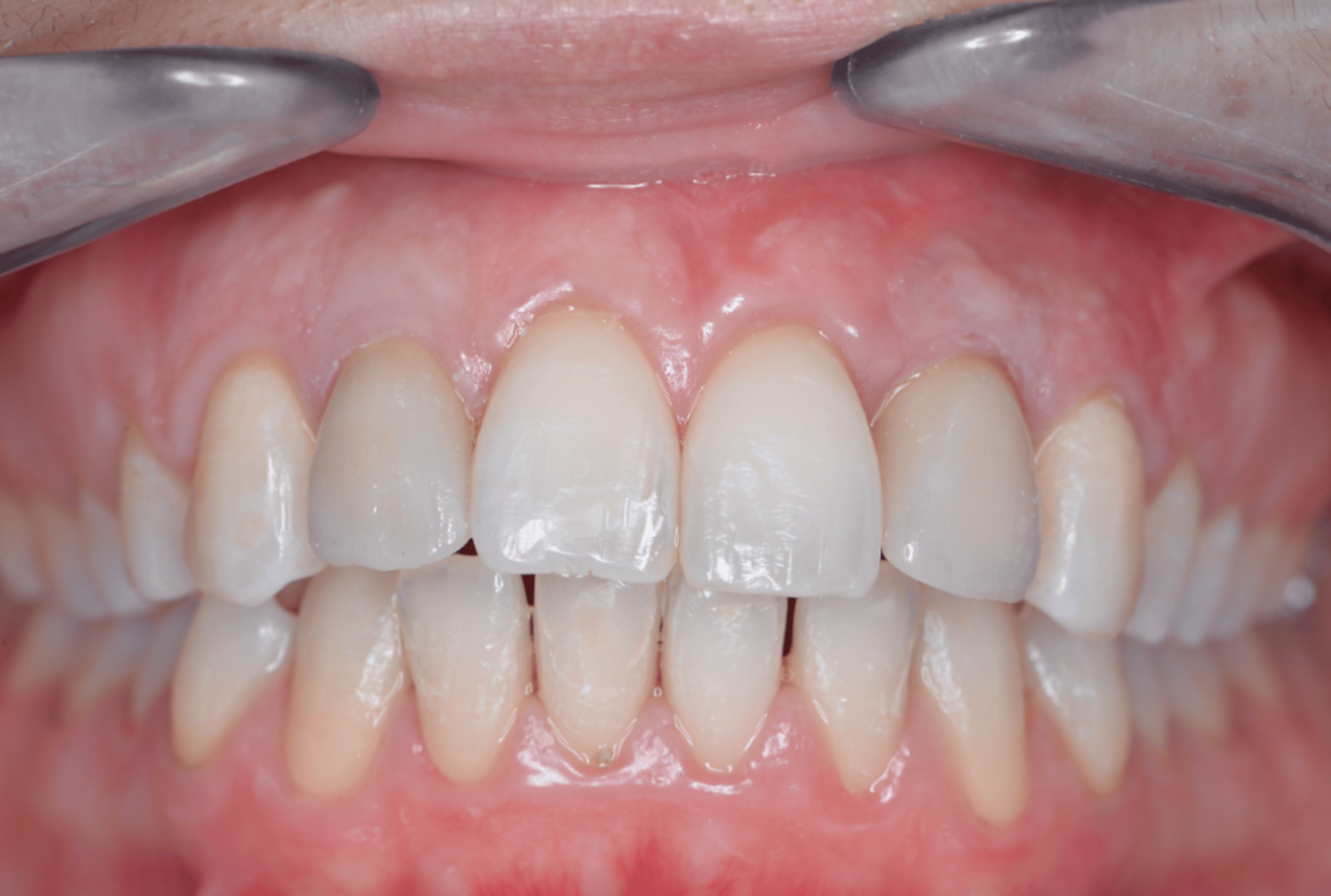
Final clinical view showing the definitive prosthetic restoration supported by both implants.

At four years of follow-up, the implants demonstrated stable function, appropriate crown proportions, and satisfactory esthetic integration. The Pink Esthetic Score (PES) and the White Esthetic Score (WES) were used to quantitatively assess the esthetics of the rehabilitation [11]. Overall values were PES13 and WES6 for tooth number 1.2 and PES 11 and WES 6 for tooth number 2.2. At seven years post-implant placement, clinical examination revealed harmonious peri-implant soft tissues and stable marginal conditions. Radiographic evaluation confirmed long-term maintenance of regenerated bone up to the interproximal bone peaks (Figure 6). The patient was extremely satisfied with the result.

## Discussion

Reconstruction of alveolar ridge defects following implant failure represents one of the most challenging scenarios in implant and periodontal therapy. Compared with primary implant sites, retreatment areas are often characterized by residual inflammation, altered vascularization, scar tissue formation, and reduced regenerative potential, all of which may negatively influence the predictability of hard and soft tissue regeneration [11-12]. In addition, implant failure in the esthetic zone frequently requires not only restoration of bone volume but also long-term maintenance of peri-implant soft tissue stability and prosthetically guided implant positioning.

Various surgical approaches have been proposed to manage vertical and horizontal ridge deficiencies, including guided bone regeneration with biomaterials and barrier membranes [13]. While these techniques have demonstrated clinical success, their predictability remains limited, particularly in non-contained defects and in sites with compromised local biology [14-16]. In addition, slowly resorbing biomaterials may interfere with early wound healing, angiogenesis, and bone remodeling, potentially complicating regenerative outcomes, especially in retreatment cases previously affected by infection or peri-implantitis [17-18].

The present case illustrates a staged, biologically driven approach using exclusively autogenous bone according to the Khoury technique [9-10]. Although the technique itself is well established and widely documented in the literature, reports with extended follow-up in previously failed esthetic implant sites remain relatively limited. The rationale for selecting this method was based on the need to restore vital bone in a site characterized by severe three-dimensional bone loss and compromised regenerative conditions, given the patient's age. By using thin cortical bone plates combined with autogenous particulate bone, the technique allows the creation of a stable regenerative compartment while promoting revascularization and progressive bone remodeling. Previous histologic studies on autogenous cortical plate augmentation procedures have demonstrated the formation of well-vascularized regenerated bone with ongoing remodeling over time, supporting the biologic rationale of this approach [19]. However, no histologic evaluation was available in the present case, and therefore conclusions regarding the quality of regenerated bone remain limited to clinical and radiographic observations.

Several biological principles critical to successful regeneration were respected: primary wound closure, adequate blood supply, space maintenance, and graft stability. The use of autogenous bone eliminates the risk of delayed resorption or foreign body reaction associated with some biomaterials and supports faster remodeling into vital bone. These aspects are particularly relevant in retreatment scenarios, where impaired healing capacity and altered bone physiology may compromise outcomes. At the same time, autogenous bone harvesting is associated with increased surgical morbidity compared with the use of biomaterials alone. Consequently, less invasive regenerative approaches based on xenografts or allografts may represent valid alternatives in many clinical situations. The choice of regenerative strategy should therefore be individualized according to defect morphology, patient-related factors, esthetic demands, and clinician experience.

In the present case, implant placement was possible after a four-month healing period, with regenerated bone capable of supporting implants in the correct prosthetic position. Long-term follow-up demonstrated stable peri-implant hard and soft tissues up to seven years after implant placement, suggesting that the regenerated bone maintained its volume and functional integrity over time.

It is important to acknowledge the limitations of this report. This is a single-case retrospective evaluation, and the outcomes cannot be generalized. Quantitative measurements of bone gain were not standardized due to the nature of the case. In addition, no direct comparison with alternative regenerative strategies was performed. Nevertheless, the extended clinical and radiographic follow-up may provide useful clinical insight into the management of severe implant failure-associated defects in the esthetic zone.

Within these limitations, the present report supports the concept that, in cases of implant failure associated with severe bone loss, a staged regenerative approach aimed at restoring vital bone may offer predictable and stable outcomes. The Khoury technique represents one of several available options and should be considered in the context of patient-specific risk factors, defect morphology, and clinician experience.

## Conclusions

Within the limitations of this single case report, staged autogenous reconstruction according to the Khoury technique allowed successful rehabilitation of a severely compromised esthetic implant site following implant failure. The seven-year clinical and radiographic follow-up suggests stable hard and soft tissue conditions over time. Although no conclusions regarding superiority over alternative regenerative approaches can be drawn, this report highlights the potential role of autogenous bone reconstruction in selected complex retreatment scenarios.
